# The Room-Temperature Chemiresistive Properties of Potassium Titanate Whiskers versus Organic Vapors

**DOI:** 10.3390/nano7120455

**Published:** 2017-12-19

**Authors:** Alexey S. Varezhnikov, Fedor S. Fedorov, Igor N. Burmistrov, Ilya A. Plugin, Martin Sommer, Andrey V. Lashkov, Alexander V. Gorokhovsky, Albert G. Nasibulin, Denis V. Kuznetsov, Michail V. Gorshenkov, Victor V. Sysoev

**Affiliations:** 1 Laboratory of Sensors and Microsystems, Yuri Gagarin State Technical University of Saratov, 77 Polytechnicheskaya str., Saratov 410054, Russia; alexspb88@mail.ru (A.S.V.); glas100@yandex.ru (I.N.B.); ilyaplygin@mail.ru (I.A.P.); avlashkov@sstu.ru (A.V.L.); algo54@mail.ru (A.V.G.); 2 Laboratory of Nanomaterials, Skolkovo Institute of Science and Technology, Skolkovo Innovation Center, 3 Nobel str., Moscow 143026, Russia; a.nasibulin@skoltech.ru; 3 Department of Functional Nanosystems and High-Temperature Materials, National University of Science and Technology MISiS, 4 Leninskiy pr., Moscow 119991, Russia; dk@misis.ru (D.V.K.); mvgorshenkov@gmail.com (M.V.G.); 4 Institute of Microstructure Technology, Karlsruhe Institute of Technology, 1 Hermann-von-Helmholtz Platz, 76344 Eggenstein-Leopoldshafen, Germany; martin.sommer@kit.edu; 5 Department of Applied Physics, Aalto University, Puumiehenkuja 2, 00076 Aalto, Finland

**Keywords:** potassium titanate, whisker, gas sensor, multisensor array, gas analysis, organic vapors

## Abstract

The development of portable gas-sensing units implies a special care of their power efficiency, which is often approached by operation at room temperature. This issue primarily appeals to a choice of suitable materials whose functional properties are sensitive toward gas vapors at these conditions. While the gas sensitivity is nowadays advanced by employing the materials at nano-dimensional domain, the room temperature operation might be targeted via the application of layered solid-state electrolytes, like titanates. Here, we report gas-sensitive properties of potassium titanate whiskers, which are placed over a multielectrode chip by drop casting from suspension to yield a matrix mono-layer of varied density. The material synthesis conditions are straightforward both to get stable single-crystalline quasi-one-dimensional whiskers with a great extent of potassium replacement and to favor the increase of specific surface area of the structures. The whisker layer is found to be sensitive towards volatile organic compounds (ethanol, isopropanol, acetone) in the mixture with air at room temperature. The vapor identification is obtained via processing the vector signal generated by sensor array of the multielectrode chip with the help of pattern recognition algorithms.

## 1. Introduction

Environmental monitoring requires sensors whose characteristics combine a long-term usage, mobility, and no special probe extraction [1,2,3]. These requirements are fully addressed by chemiresistive gas sensors, which are primarily based on metal oxide structures [4] since pioneer introduction by Seiyama [5]. These sensors are known to be robust under a long-term operation with rather high response, which is matured from an advanced receptor function due to existence of number of surface adsorption sites and a transduction function that is based on nano-dimensionality of characteristic percolation contacts at the polycrystalline samples. However, these sensors should be activated by quite high temperatures, up to 400 °C [6,7], or by UV radiation [8,9], to allow for electron exchange between local states in the semiconductor gap and conductance band, as well as surface reactions. However, many applications, like mobile gas-analytical units [10], need sensors to operate just at a room temperature. The reduction of power consumption is also a target. So, there is a great demand in searching the chemiresistive materials that can work under such conditions [11].

A choice of materials to reply to this task is limited so far mostly to carbon-based materials such as carbon nanotubes [12,13], graphene [14,15] and its derivatives [16,17], other (quasi-) two-dimensional (2-D) layers, like MoS_2_ [18], oxide-derived structures based on indium tin oxide [19], Fe_2_O_3_ [20], SnO_2_ [21], ZnO [22], and In_2_O_3_ [23], which is grown in architectures of nanowires or nanotubes, and ionic conductors like ionic liquids [24] and solid electrolytes [25,26]. Except for UV radiation, these and other nano-structured materials could be further self-activated by Joule heating under application of electric field [27,28] to gain a chemiresistive effect. However, titanates have not been explored for designing gas sensors though they are well known catalysts to have both electronic and ionic conductivity [29], which makes it promising to try out their chemiresistive properties. A particular advantage of this material is its layered structure with the layers built from [TiO_6_] octahedral units and mobile metal/hydroxonium ions that are located in the interlayer space to maintain electroneutrality what determines its rather good conductivity even at room temperature. Therefore, recently we have touched gas-influenced impedance of these potassium polytitanate structures at advanced temperatures [30]. Moreover, one of the essential properties of this material is a possibility to be grown under a variety of morphological forms [31,32,33,34]. Depending on a ratio of precursors and sintering temperature and/or further treatment, one can tune the crystalline structure and composition of the final product. For instance, whisker-like morphology is usually observed for potassium titanates that are prepared from TiO_2_/K_2_O mixtures with a molar ratio greater than 4:1 and sintered at temperatures of higher than 900 °C [35,36,37]. Such a quasi-one-dimensional morphology is frequently mentioned in other above-listed works to favor the gas sensitivity at room temperature because of high surface-to-volume ratio. Moreover, the whiskers’ architecture, exactly its macroscopic dimension in direction along the whisker’s axis, allows one expecting a stable functionality [38]. Therefore, in this work we have approached the chemiresistive properties of potassium titanate whiskers under room temperature for development of new gas-analytical multisensor array chips.

## 2. Results and Discussion

### 2.1. Composition and Structure of Potasium Titanate Whiskers

Sintered potassium titanate whiskers are suggested to contain two phases, К_2_Ti_4_O_9_ and H_2_Ti_8_O_17_ (see Supplemental), as it has been argued earlier [35,39]. The first phase possesses a layered structure whereas the second one where a number of Ti atoms in gross formula greater than 5 is characterized by tunnel-like structure [40,41]. Though, the layered structure is less stable it is assumed to have higher ion exchange capacity [42]. It is worth noting that the molar TiO_2_/K_2_O ratio in the precursor phase according to diagram of K_2_O-TiO_2_-K_2_O·*n*TiO_2_ (*n* = 2, 4, 6) system [43] primary defines the output product phase after the synthesis. In accordance with the diagram, the structures of K_2_Ti_4_O_9_ are appeared following annealing at temperature greater than 1000 °C when the TiO_2_/K_2_O ratio in the precursor is about 4:1 [44]. In addition, H_2_Ti_8_O_17_ related structures, like K_2_Ti_8_O_17_, are expected to appear following a hydrolysis of K_2_Ti_4_O_9_ at temperatures in the range of 250 °C–500 °C [41,45].

Sonification in acid media yields debundled whiskers and modifies drastically the specific surface of the material. According to BET results, the untreated agglomerates of titanate whiskers are characterized by specific area of 5–9 m^2^/g, which increases up to 170–250 m^2^/g following five runs of the treatment (see Supplemental). These changes are accompanied by a reduction of pH value from 12–14 to 6–7, due to both a neutralization of residual base and exchange processes between potassium ions from the titanate interlayer space and hydrogen ions from the solution. The extraction of the potassium ions has been already shown to gradually follow the decrease of pH value [44]. The energy-dispersive X-ray spectroscopy (EDX) elemental analysis of the titanate whiskers prior and after the sonification in acid further indicates a significant reduction of [K] atomic content from ca. [Ti]/[K] = 4 down to ca. [Ti]/[K] = 9–15 what suggests the whiskers to appear mainly at potassium titanate phase with increased amount of protons. Moreover, we may attribute the increase of specific surface in the material to exfoliation of the titanate whiskers facilitated by the ion-exchange process, which provokes the reduction of layers between-in electrostatic forces as potassium ions are replaced by hydrogen ones. Further centrifugation of the obtained material allows one to separate whiskers as the final product for placing over chip.

**Figure 1:**
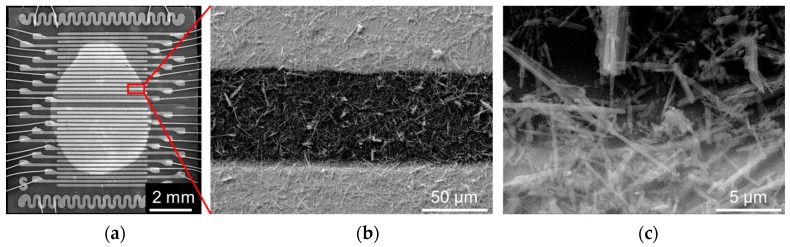
The scanning electron microscope (SEM) images of multielectrode chip equipped with titanate whiskers: (**a**) image of the whole chip; (**b**) the matrix whisker layer confined between two electrodes at single sensor segment; and, (**c**) whiskers at the electrode edge.

According to results of scanning electron microscope (SEM) inspection, the finally fabricated potassium titanate whiskers are characterized by mean length of 10–30 μm and a thickness of about 0.5–1 μm. When placed over the multielectrode chip surface for resistance measurements, they constitute a matrix mono-layer with multiple interconnections, as shown in Figure 1. The whiskers distribution over the surface is not spatially homogeneous, which leads to variations in functional characteristics of the chemiresistor segments in the array as discussed in Section 2.2.

**Figure 2:**
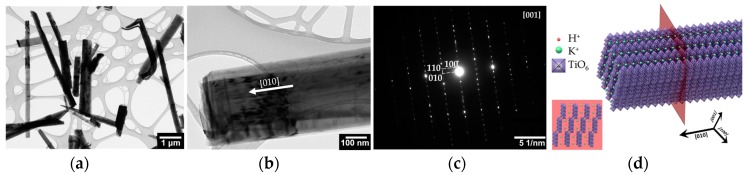
TEM images of titanate whiskers: the typical bright field image of (**a**) the whiskers; (**b**) single whisker; (**c**) selected area electron diffraction (SAED) image; (**d**) model of the titanate whisker structure.

TEM images of potassium titanate whiskers are presented in the Figure 2a,b. The images support the quasi-one-dimensional morphology of the titanate structures. The SAED results that are obtained from the particular exemplary whisker have revealed its single-crystal structure, as it is shown in Figure 2c. These single crystals are characterized by monoclinic crystal symmetry, C2/m (12) space group, with the *d*-values of 3.77 Å, 3.60 Å, 12.40 Å, and 6.24 Å correspondingly for (010), (110), (100), and (200) planes. Moreover, the reflections are clearly identified for higher-order planes.

The whisker is oriented along [010] axis, which is proven to be a preferential growth direction for other titanate structures, like hexatitanate [46,47], whose appearance from K_2_Ti_4_O_9_ phase is to be observed at temperatures that are higher than 1100 °C [48]. A preferential crystal orientation seems to appear due to the energy differences in K-O and Ti-O bondings at TiO_6_ octahedron under synthesis conditions that mainly favor splitting between (100) and (010) planes to form quasi-one-dimensional structure at a preferential [010] axis [31].

Our SAED data cannot be fully matched to crystal structure of K_2_Ti_4_O_9_ (ICDD, 00-032-0861), H_2_Ti_8_O_17_ (ICDD, 00-036-0656), or other titanate phases available in ICDD database. Though, we can indicate a good match for the *d*-values in direction of [010] axis that is observed in our structure when compared to the mentioned phases. At the same time, the *d*-values corresponding to reflections observed at [100] axis are greatly reduced. This hints that our whiskers are akin composed of layers of [TiO_6_] octahedral units with interlayer space to be quite reduced possibly due to intensive replacement of potassium ions by hydrogen ones in accordance with other characterization data described above.

Based on these findings we propose the model of whiskers drawn in Figure 2d, which reflects its layered structure constituted by octahedral units with in-between potassium and hydrogen ions. Here, several collinear edge-sharing TiO_6_ octahedra compose the 2D zigzag-like sheets [30]. This model suggests that potassium and hydrogen ions could move at least in direction along the whisker length in channels formed between adjacent [TiO_6_] layers.

### 2.2. Chemiresistive Properties of Potassium Titanate Whiskers 

**Figure 3:**
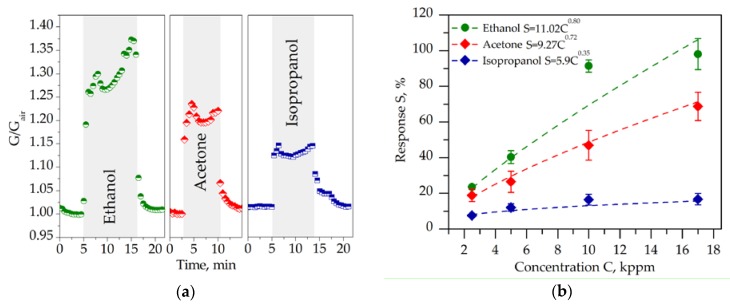
The gas response of the multisensor chip based on titanate whiskers towards organic vapors in the mixture with lab air at room temperature: (**a**) the typical evolution of the exemplary sensor segment conductance, *G*, under appearance of the vapors, ~5 kppm concentration, normalized by the conductance in air, *G_air_*; (**b**) the dependence of the chemiresistive response, *S,* to organic vapors on their concentration, *C*.

We have studied gas-sensing performance of the chip based on matrix layer of the potassium titanate whiskers by exposing it towards the three organic vapors, ethanol, acetone, and isopropanol, in the mixture with air. In the presence of organic vapors the conductance of all the sensor segments grows up as shown for example in Figure 3a when vapors were added to air at 5 kppm concentration. The effect is found to be reproducible (see Supplemental). The most significant median response, *S*, is observed to ethanol vapors to be approx. 40.4 ± 3.6%, while that to acetone and isopropanol is 26.5 ± 5.95% and 12.0 ± 2.1%, respectively. The higher concentration of vapors results in larger reversible enhancing of conductance as shown in Figure 3b. The response-to-concentration curves are well fit by a power law *S* = *aC^b^*, where *b* is equal to 0.80, 0.72 and 0.35, respectively, for ethanol, acetone, and isopropanol, in accordance with the behavior of typical Freundlich isotherm. It is clear that acetone and ethanol are much more sensitive for the titanate when compared to isopropanol. The reversible chemiresistive response is observed to all of the three test vapors.

It is worth noting that the conductance of these complex titanate structures is matured from both the ionic and electronic components due to mobile K^+^, H^+^, and electrons, respectively. In ordinary air conditions, the whisker’s surface is covered by oxygen and hydroxyle/humidity species [49] chemisorbed firstly at defects such as oxygen vacancies to form oxygen ions or hydroxide radicals on the surface. These adspecies induce the space charge region near the titanate surface to reduce primarily the electron concentration: a number of free electrons are drawn out the conduction band into localized states that are generated by the adspecies. Except for depletion of at-surface layer in the titanate the appeared surface potential might substantially affect a mobility of ions through channels within the titanate layers. On top of that, the conductance of whiskers’ layer depends on potential barriers appeared at the whiskers contacts, as frequently observed in such matrix layers [50]. These barriers further reduce the bulk conductivity of single whiskers.

**Figure 4:**
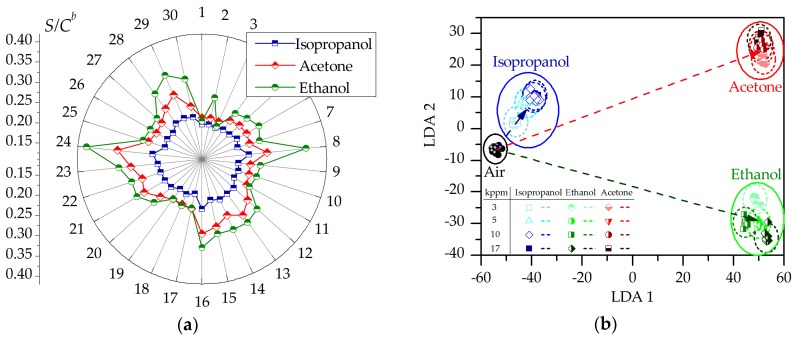
(**a**) The distribution of sensor segment, # 1-30, responses, *S,* normalized by *C^b^* value taken from *S(C)* power law dependence given in Figure 3b, only the mean *S/C^b^* values of each sensor segment are shown for clarity; (**b**) the projection of the multisensor array signal versus the organic vapors, 3–17 kppm concentration, by Linear Discriminant Analysis (LDA) to the first two component coordinate system, the points indicate the test multisensor responses to vapors, ellipses frame the corresponding LDA space zones built under 0.95 confidence level with the help of sampling of 20 training vector points, the arrows display the “transfer” of vector signals from air-related cluster to the vapor-related clusters.

We believe that the appearance of organic vapors that are accompanied by chemisorption of the corresponding adspecies over the surface of whiskers first decreases its coverage by oxygen and humidity related adspecies and returns the at-surface captured electrons back to the titanate conductance band due to the surface reactions. Second, the reduction of surface electric potential leads to a lowering of the barriers that appeared both between whiskers and at the whiskers-electrode interface. The mentioned processes lead to enhancing the titanate layer conductance in dependence on the vapor type. This response is enhanced for the segments of whisker layer, which have lower conductance recorded in pure lab air. This might be primarily due to lower density of the whiskers at these segments. So, because of spatial variation of the matrix layer each sensor segment in the array has a specific value of not only the conductance value but also the gas response [51]. We highlight this discrepancy in the Figure 4a, yielding a radar distribution of sensor segment responses to the vapors. In order to make clear the vapor-related differences in the distribution to be independent on vapor concentration, we plot here the mean values of *S’* = *S/C^b^* for each sensor segment of the array taken for all of the experimental responses *S* that are measured at all of the test vapors concentrations *C*. As one can see from the radar plot, the patterns of the distribution observed at various vapors are quite different. The nature of this remarkable difference comes obviously from the differences in interaction of vapor molecules with other chemisorbed adspecies and the titanate layer itself, which should be further clarified.

But, despite on some differences in the response of the titanate whiskers to the test organic vapors, it is impossible to distinguish them if the gas concentration is unknown. Therefore, to approach the selectivity, we have considered the vector signal of the whole multisensor array located at the chip whose components are conductances of each sensor segment. A difference of vapor interaction with the titanate whiskers’ layer yields an effect on the observed distribution of segment responses over the array, which can be extracted by pattern recognition techniques, like LDA. Prior to analysis the conductance array vectors have been normalized by a median value *G_med_* taken over the array:

(1) {G1…Gi…Gk}n→{G1Gmed…G1Gmed…G1Gmed}n

where *G_i_* is the conductance of sensor segment *i* from the chip array, *k* is the number of segments in the array, *n* is the number of test gases including pure air.

Indeed, when we transfer the vector signals of the studied multisensor array versus the three test vapors appeared at all of the concentration range into the LDA phase space we could see their clear separation, as shown in the Figure 4b. According to general approach, we have built primarily LDA model ellipses based on sampling of 20 vector points to each gas, and tested other vector signals shown as points in the LDA diagram versus these ellipses. The shown projection is given in the coordinate system of first two of three LDA components. The presented data points tend to be assembled into clusters in frames of ellipses. The vector signals group according to different vapor concentrations to be assembled into larger clusters that are related to each test gas with mean distance between gravity centers equal to ~80 un. It is clear that the obtained cluster distinction makes possible to distinguish the vapors of various origin independent on a concentration. It is worth noting that further advancing of selectivity of the developed titanate whisker–based chip is possible via introduction of foreign additives [52] and by other [53] methods of differentiation of the layer’s functional properties over the chip.

## 3. Materials and Methods

Potassium titanate whiskers have been synthesized according to the following route. Primarily, we have synthesized the precursor, potassium polytitanate, by hydrothermal method [44]. The titania (anatase) powder has been mixed with potassium hydroxide and distilled water in 2:5:7 mass-ratio and placed into stainless steel crucible to be treated at 110 ± 10 °C for 24 h. The obtained product has been washed in distilled water using 100 mL of water for each 10 g of the synthesized material, and further dried in desiccator at 85 ± 5 °С for 12–14 h until its mass has been stabilized. The synthesized material is characterized by quasi-amorphous structure and developed morphology, which has been confirmed by X-ray diffraction (XRD, Thermo Scientific ARL X’TRA, diffractometer, Ecublens, Switzerland), scanning electron microscopy (SEM, Tescan VEGA 3, Brno, Czech Republic) inspection, and Secondary Neutral Mass Spectrometry (SNMS, Leybold-Heraeus, Koeln, Germany); see Supplemental. Further, we have calcined this product at 1050 ± 10 °С in oven for 1 h in air atmosphere. Heating has been applied with a rate of 10 °/min. The oven has been let to cool down for 10–12 h afterwards. The sintered material, which is represented by a mixture of potassium titanate whiskers bundles and a glass phase, has been coarsely grinded and milled in agate bowl for 3 min using mortar grinder (Fritsch Pulverisette 2, Idar-Oberstein, Germany). We have added distillated water to the obtained powder to get 10% mass slurry. This slurry has been characterized by pH that is equal to 10–12. In order to neutralize the residual base, the 30% HCl solution has been added dropwise to the slurry until the value of pH equal to 5–6 is reached. Then the suspension has been treated by sonification at the power of 1 kW, 20 kHz (Hielscher UIP1000hd, Teltow, Germany) for 2 min. The sonification has enhanced the pH of solution up to 7–8, which is facilitated by release of residual base and by hydrolysis of the titanates. We have repeated the described treatment several times until the pH has been stabilized at 6–7.

The final suspension has been centrifuged to separate the solid phase to be further intensively stirred with added 1000 mL of distil water to each 170 ± 10 g of the slurry. This procedure has been repeated few times to wash away the residual KCl. After the last washing, the powder of potassium titanate whiskers has been dried at temperature of 90 °С for 10–12 h to be utilized for making a gas-analytical multisensor chip afterwards.

The material surface area has been evaluated by Brunauer-Emmet-Teller (BET) method with the help of Quantachrome Nova2200 analyzer (Boynton Beach, FL, USA). Morphology of the powder and chip surface have been inspected with SEM (JSM-6610 JEOL, Tokyo, Japan) at 20 kV coupled with energy-dispersive X-ray spectroscopy (EDX, X-MAX, Oxford Instruments NanoAnalysis, Oxfordshire, UK). Transmission electron microscopy (TEM) and selected area electron diffraction (SAED) studies have been conducted using such instruments as JEOL JEM 1400 (Tokyo, Japan) under an accelerating voltage of 120 kV and FEI Tecnai G2F20 S-Twin TMP (Eindhoven, The Netherlands), under accelerating voltage of 200 kV. XRD inspection has been utilized at all of the stages of material synthesis to assess its crystal phase using ARL X’TRA with CuKα (λ_CuKα_ = 0.15412 nm) in the range of 2θ from 5° to 60°. The results are detailed in Supplemental.

**Figure 5:**
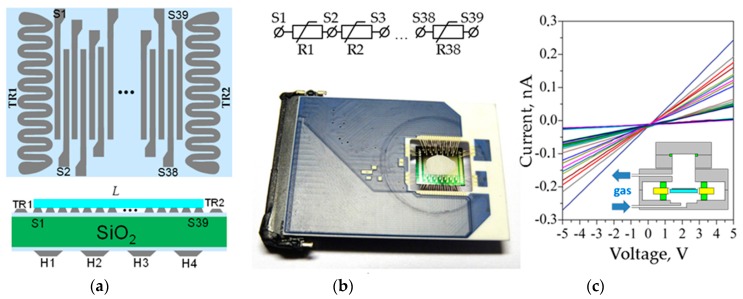
The multisensor array chip employed to study the chemiresistive properties of potassium titanate whiskers: (**a**) the scheme of front side and cross-section, S1-S39 are the numbered electrodes, *L* is the gas-sensitive layer, TR1, TR2 are thermoresistors, H1-H4 are the meander heaters; (**b**) the equivalent electrical scheme and photo of the chip wired into 50-pin ceramic housing (Siegert); (**c**) *I-V* characteristics of the sensor segments in the array measured under lab air without addition of organic vapor; and, the insert is the scheme of chamber housing the chip.

As a sensor platform, we have employed Si/SiO_2_ substrate, 10 × 10 mm^2^, with sputtered multiple Pt electrode strips, about 100 μm width, 4 mm length distanced at 80 μm, up to 39 in number [54]. The substrate front side is equipped with two Pt thermoresistors of ca. 1 μm, while four Pt heaters are realized at the back side; see Figure 5a. These heating facilities have not been employed in this study.

To equip the chip with the whisker layer, we have prepared a set of dispersions of the corresponding powder in distilled water under different concentrations to evaluate/adjust the optimal thickness of the gas sensitive layer. The dispersions’ concentration has been chosen to be 5, 0.1, 0.002, and 0.0002% mass based on preliminary investigations [55]. The 0.01 mL droplet of the dispersion has been cast onto the SiO_2_ substrate surface over the multielectrodes, and dried at room temperature for 24 h. In case of 0.1% mass suspension an almost mono-whisker thick matrix layer of the lowest density has been obtained (See Supplemental).

Each pair of electrodes of the chip array confines a segment layer (Figure 5a,b) whose conductance has been measured by custom-made electronics setup. This setup has been described in details earlier [56]. Briefly, it has been based on NI-6259 acquisition board whose measurement inputs have been multiplexed to the chip electrodes via low-noise current preamplifier SR570. All of the setup has been driven by PC. Prior to all of the measurements, we have checked *I-V* characteristics of each layer segments in the array located within the chip. Only the layer segments, which exhibited linear *I-V*s, have been measured to ensure the absence of significant potential barriers at electrode interfaces. The examples of recorded *I-V*s are drawn in Figure 5c. In order to measure the *G(t)* curves, each sensor segment of the chip has been sequentially read out by the acquisition board via multiplexor.

In order to perform gas-sensing measurements, the chip is placed into stainless steel chamber, of less than 1.75 mL internal volume available for gas probe, whose cross section is drawn in inset of Figure 5c. Further details could be found in [57]. We have considered here as test gases the vapors of ethanol, acetone and isopropanol to be mixed with lab air of normal humidity level equal to 35–45 rel. %. All of the gas-sensing measurements have been conducted under a gas flow mode, with the rate of 100 sccm. The chip has not been heated and functioned at the room temperature. The gas response of each sensor segment of the chip is calculated according to

(2) $$S=\frac{\left|G_{gas}-G_{air}\right|}{G_{air}}$$

where *G_gas_* and *G_air_* are the segment conductance under test gas exposure (mixture of organic vapors with air) and in pure lab air, correspondingly.

In order to realize the vapor discrimination, we have collected the conductance of all the sensor segments confined between the electrodes at the chip, and considered it as a multisensor vector signal. This signal has been processed by LDA to transfer it to artificial phase space where the vector signals are grouped into clusters depending on specific vapor features, i.e., type of vapors, at the maximum possible distance between their gravity centers [58]. The dimensionality of the LDA space is equal to *N*-1, where *N* is the number of test gases plus pure air. For illustration purposes here, we employ LDA diagrams in the coordinate system of first two LDA components as the most meaningful dimensions.

## 4. Conclusions

We have evaluated the chemiresistive performance of layered potassium titanate whiskers which are synthesized by calcination of potassium polytitanate at 1050 °C. The potassium ions in the whiskers are replaced to a certain extent by hydrogen ions via further processing that leads to the reduction of inter-layer spacing and an increase of specific surface area, which might be provoked by splitting of the whiskers along their axes. The synthesized whiskers that are placed over multielectrode chip are shown to be sensitive to organic vapors, as ethanol, isopropanol, and acetone, at room temperature. The non-homogeneous matrix-like morphology of the whiskers layer seems to be responsible for the chemiresistive response of each sensor segment, which has a significant scatter over the array. This pristine fluctuation of functional properties makes it possible to build the array vector signals whose vapor-specific features could be extracted by pattern recognition algorithms, like LDA, to result in the selective identification of the vapors by the chip.

## Supplementary Materials

The following are available online at www.mdpi.com/2079-4991/7/12/455/s1, File Supplemental.
