# Supplementary material for: The Room-Temperature Chemiresistive Properties of Potassium Titanate Whiskers versus Organic Vapors
Source: Nanomaterials (Basel). 2017 Dec 19;7(12):455. doi: 10.3390/nano7120455 (PMC5746944; doi:10.3390/nano7120455)
Supplement: Supplementary file 1 [file nanomaterials-07-00455-s001.pdf]

nanomaterials

MDPI

Supplementary Materislas

The Room-Temperature Chemiresistive Properties of Potassium Titanate Whiskers versus Organic Vapors

Alexey S. Varezhnikov [1], Fedor S. Fedorov [2,*], Igor N. Burmistrov [1,3], Ilya A. Plugin [1], Martin Sommer [4], Andrey V. Lashkov [1], Alexander V. Gorokhovsky [1], Albert G. Nasibulin [2,5], Denis V. Kuznetsov [3], Michail V. Gorshenkov [3] and Victor V. Sysoev [1,3,*]

[1] Laboratory of Sensors and Microsystems, Yuri Gagarin State Technical University of Saratov, 77 Polytechnicheskaya str., Saratov 410054, Russia; alexspb88@mail.ru (A.S.V.); glas100@yandex.ru (I.N.B.); ilyaplygin@mail.ru (I.A.P.); avlashkov@sstu.ru (A.V.L.); algo54@mail.ru (A.V.G.)
[2] Laboratory of Nanomaterials, Skolkovo Institute of Science and Technology, Skolkovo Innovation Center, 3 Nobel str., Moscow 143026, Russia; A.Nasibulin@skoltech.ru
[3] Department of Functional Nanosystems and High-Temperature Materials, National University of Science and Technology MISiS, 4 Leninskiy pr., Moscow 119991, Russia; dk@misis.ru (D.V.K.); mvgorshenkov@gmail.com (M.V.G.)
[4] Institute of Microstructure Technology, Karlsruhe Institute of Technology, 1 Hermann-von-Helmholtz Platz, 76344 Eggenstein-Leopoldshafen, Germany; martin.sommer@kit.edu
[5] Department of Applied Physics, Aalto University, Puumiehenkuja 2, 00076 Aalto, Finland
* Correspondence: f.fedorov@skoltech.ru (F.S.F.); vsysoev@sstu.ru (V.V.S.); Tel.: +7-495-280-14-81 (F.S.F.); +7-8452-998626 (V.V.S.)

Received: 13 November 2017; Accepted: 11 December 2017; Published: date

A. Characterization of the precursors and the final powder

The precursor, potassium polytitanate, has been sintered and further used to prepare potassium titanate whiskers [S1]. The as-sintered precursor has been inspected by scanning electron microscopy, SEM (Tescan VEGA 3, Brno, Czech Republic), under an accelerating voltage of 15 kV. The SEM images are shown in the Figure A1. The potassium polytitanate is presented by irregularly shaped nanoparticles which are agglomerated to macro-assemblies of few micrometers.

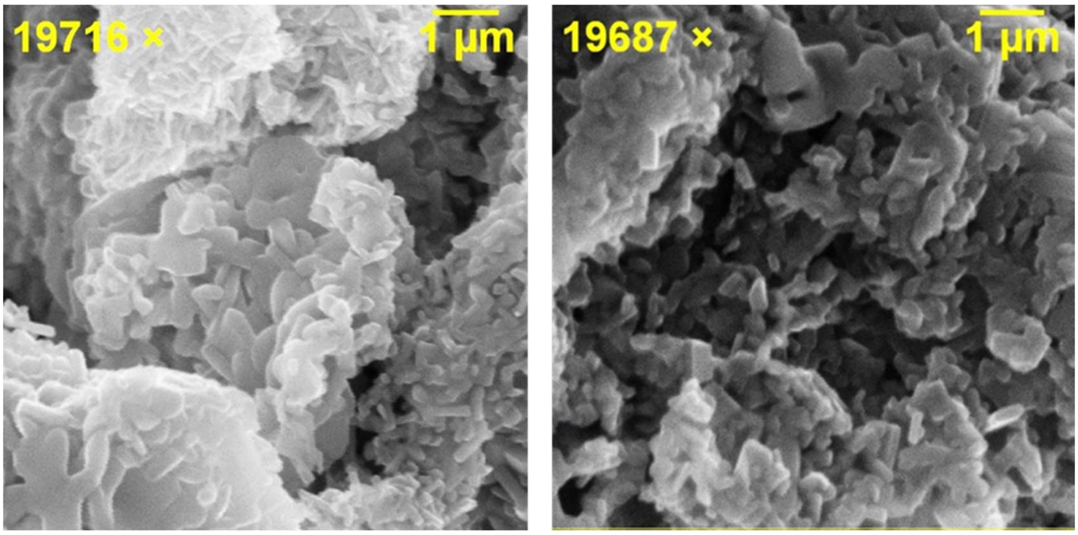

Figure A1. Scanning electron microscopy (SEM) images of sintered potassium polytitanate powder.

The calcination of the potassium polytitanate at 1050 ± 10 °C for 1 hour results in appearance of whiskers imbedded into glass phase; the SEM images are presented in the Figure A2. The images are recorded following the grinding of the obtained product.

The X-ray diffraction (XRD) of the potassium polytitanate has been carried out using ARL X'TRA diffractometer (Ecublens, Switzerland), with CuKα source of 0.15412 nm wavelength. The data are given in Figure A3. XRD results of the potassium polytitanate studies possess no evident characteristic reflections from crystalline planes that is caused by irregular agglomerates to appear in quasi-amorphous state. The calcination leads to formation of two phases, $H_2Ti_8O_{17}$ and $K_2Ti_4O_9$.

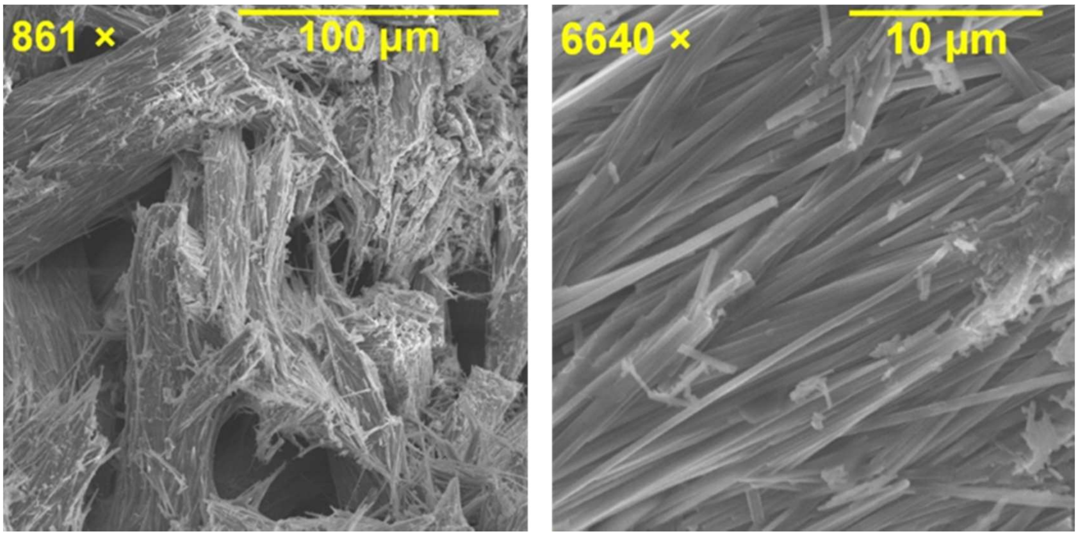

Figure A2. SEM images of sintered potassium titanate whiskers embedded into glass phase.

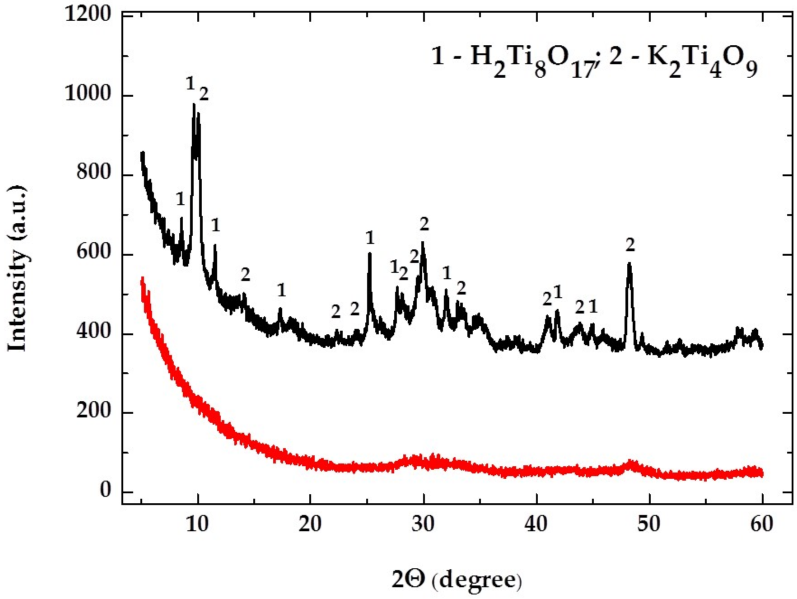

Figure A3. X-ray diffraction (XRD) patterns of as-prepared potassium polytitanate (red) and of the sintered potassium titanates after calcination at 1050 °C (black).

The obtained material is represented by quasi-1D whiskers, whose width is in sub-micrometer range, while the length reaches up to hundreds of micrometers. The glass phase is related to unreacted residuals.

Further ultrasonic treatment of the grinded product in acidic media supports the removal of residual base and an increase of specific surface area. The final product is presented in the Figure A4. There are whiskers of different widths and thicknesses, which are mainly in the range of tens of nanometers while the length is in micrometer range. The sonification in acidic media results in replacement of potassium ions by hydrogen ions [S2,S3] which facilitates the increase of the specific surface of the material, as measured by BET method (Quantachrome Nova2200, Boynton Beach, FL,

USA). The increase of the surface is the most drastic for the slurry treated to get the lowest pH (6–7) value. The dependence of specific surface on the pH treatment is summarized in the Table A1.

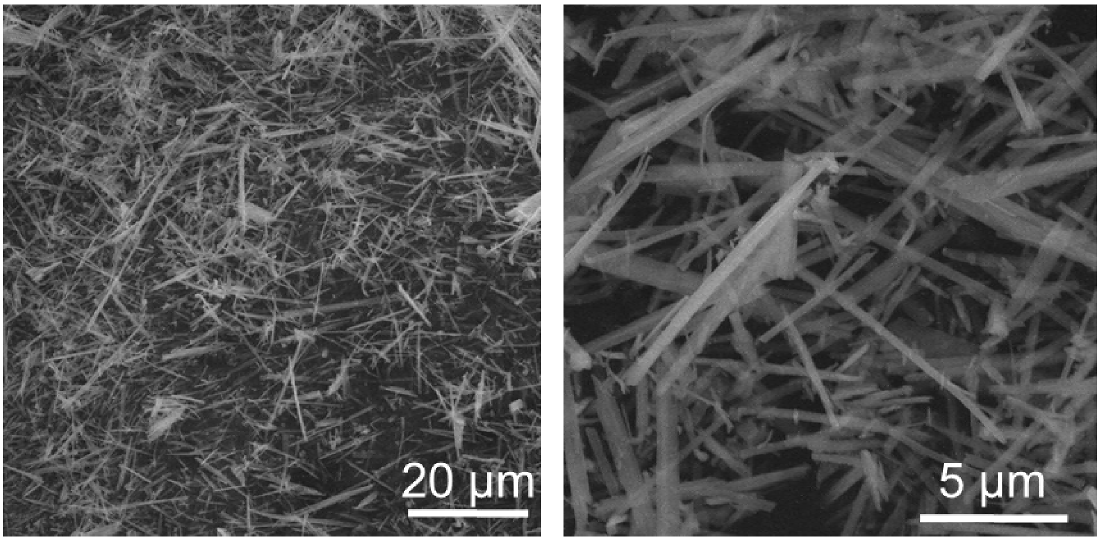

Figure A4. SEM images of potassium titanate whiskers after sonification in acidic media.

Table A1. Specific surface of the potassium titanates obtained after calcination and further milling depending on number of sonification cycles in acidic media.

No	Cycle, a.u.	pH of slurry	Specific surface, S_{sp}, m^2/g
1	-	12–14	5–9
2	3	8–9	15–35
3	5	6–7	170–250

The final whiskers are characterized by layered structure, which is supported by HR-TEM studies (Figure A5).

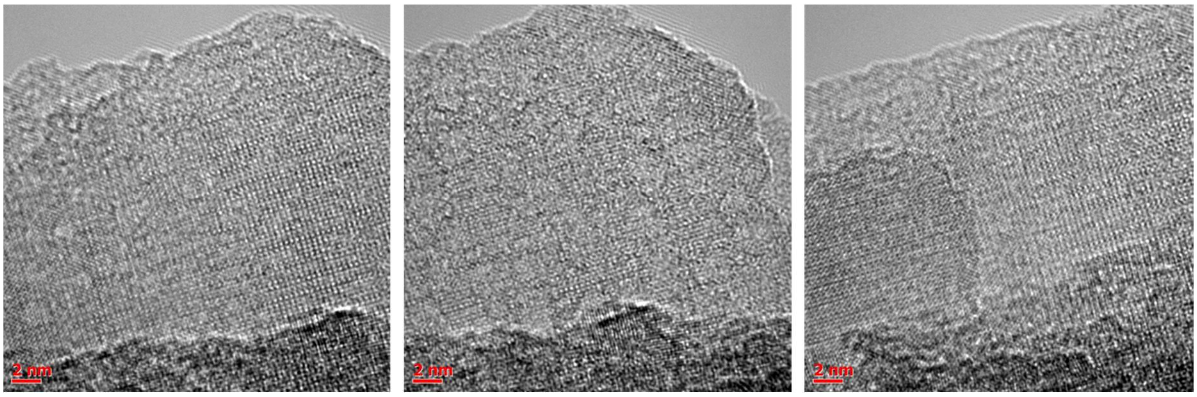

Figure A5. High resolution transmission electron microscopy (HR-TEM) images of potassium titanate whiskers.

We have performed the elemental analysis of the final titanate structures by Secondary Neutral Mass Spectrometry (SNMS) in INA 3 system (Leybold-Heraeus, Germany). In that technique, the sample is located in vacuum chamber, where its surface is bombarded by positive ions that are extracted from RF-excited argon plasma [S4]. Being induced by the ion bombardment, material is removed from the sample mainly as neutral atoms, which are ionized by electron impact when passing through the plasma. After suppression of thermal ions, a quadrupole mass spectrometer is finally used for mass analysis. Since the atoms could be excited not only from the sample, but also from the mask and a whole setup, first the spectrum was measured for the elemental composition of

the carrier. The data received after these measurements were used to correct the number of atoms that were extracted from the sample in the received spectra. The details are given in [S5–S7]. The spectrum has been recorded to analyze the elemental composition at the surface of the polytitatanes. The obtained data are summarized in the Table A2.

Table A2. Secondary Neutral Mass Spectrometry (SNMS) data related to elemental composition of potassium titanate structures.

Element	Atomic content, %
[O]	43.6
[Ti]	23.3
[K]	1.5
[S]	1.81
[Al]	0.29

From the received data [O]/[Ti] is equal to 1.9, and the ratio of [Ti]/[K] is 15.3. This further supports results of elemental analysis by energy-dispersive X-ray spectroscopy.

The additional TEM/SAED measurements of obtained whiskers been taken using FEI Tecnai G2F20 S-Twin TMP (Eindhoven, The Netherlands) instrument applying accelerating voltage of 200 kV. Line resolution is 0.14 nm. The SAED results (Figure A6) that were taken in a repeated set of characterization measurements, reveal d-values of 3.65, 3.49, 11.79 and 5.89 Å, correspondingly for (010), (110), and (100), (200) planes. The whisker is oriented along [100] axis.

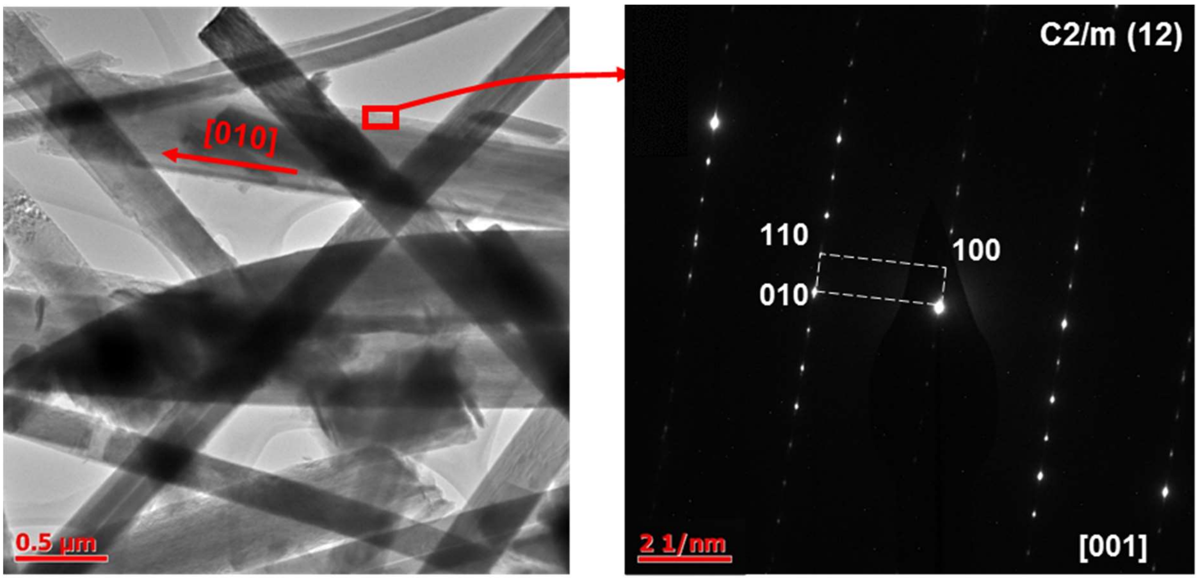

Figure A6. TEM/SAED images of potassium titanate whiskers.

Our FFT results that were obtained at HR-TEM image (Figure A7) additionally confirm the d-values calculated to be 3.74, 3.48, 9.47, 4.73 Å, for the planes (010) and (110), (100), (200) planes. Moreover, the d-values for higher-order planes are 2.41, 1.74, 1.87, 2.37, 2.93, and 2.00 Å in case of (310), (220), (020), (400), (210), (410) planes, respectively.

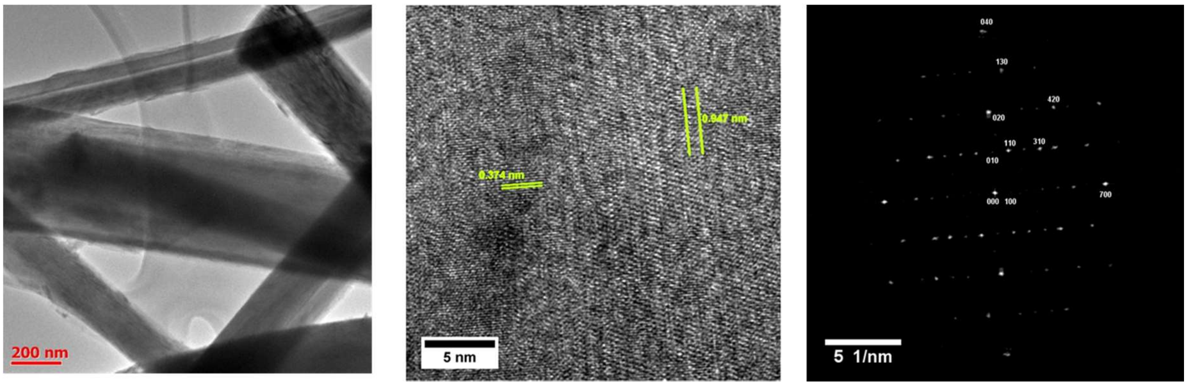

Figure A7. TEM images of potassium titanate whiskers and corresponding results of FFT performed in the HR-TEM image.

B. Sensor preparation

We have optimized the suspensions based on the final product to get almost a single-whisker layer, depending on the concentration of the whiskers in solutions. A set of dispersions has been prepared where whisker concentration is varied from 5.0% mass to 0.0002% mass. Dispersion has been drop-casted on the Si/SiO_2 wafer and let dry at room temperature. SEM inspection suggests that employing suspensions with a concentration of whiskers lower than 0.002% does not lead to the formation of contacts to support the current flow between the electrodes (Figure B1).

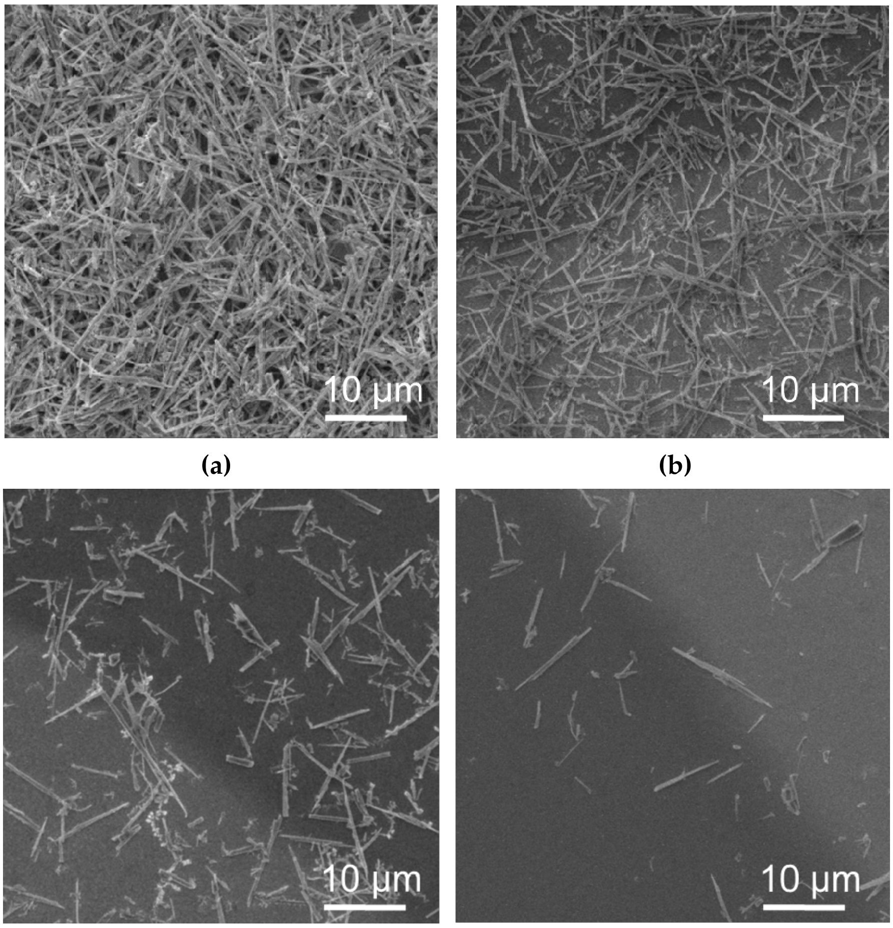

(c) (d)

Figure B1. SEM images of potassium titanate whiskers drop-casted onto Si/SiO_2 substrate from suspensions with concentration (% mass): (**a**) 5%; (**b**) 0.1%; (**c**) 0.002%; and, (**d**) 0.0002%.

Utilization of 5% suspension results in appearance of layer with thickness of several whiskers. At this morphology some whiskers are limited to be exposed to gas environment. So, we have utilized the suspension of 0.1% mass for the chip preparation.

C. Gas-sensing characteristics

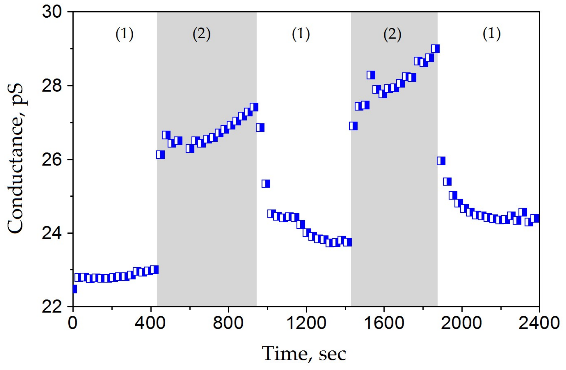

Figure C1. Typical change of the median conductance of the titanate whisker layer under appearance of the isopropanol vapors, 5 kppm, in the mixture with air, (1), (2) denote pure lab air and mixture of air with isopropanol vapors, respectively.

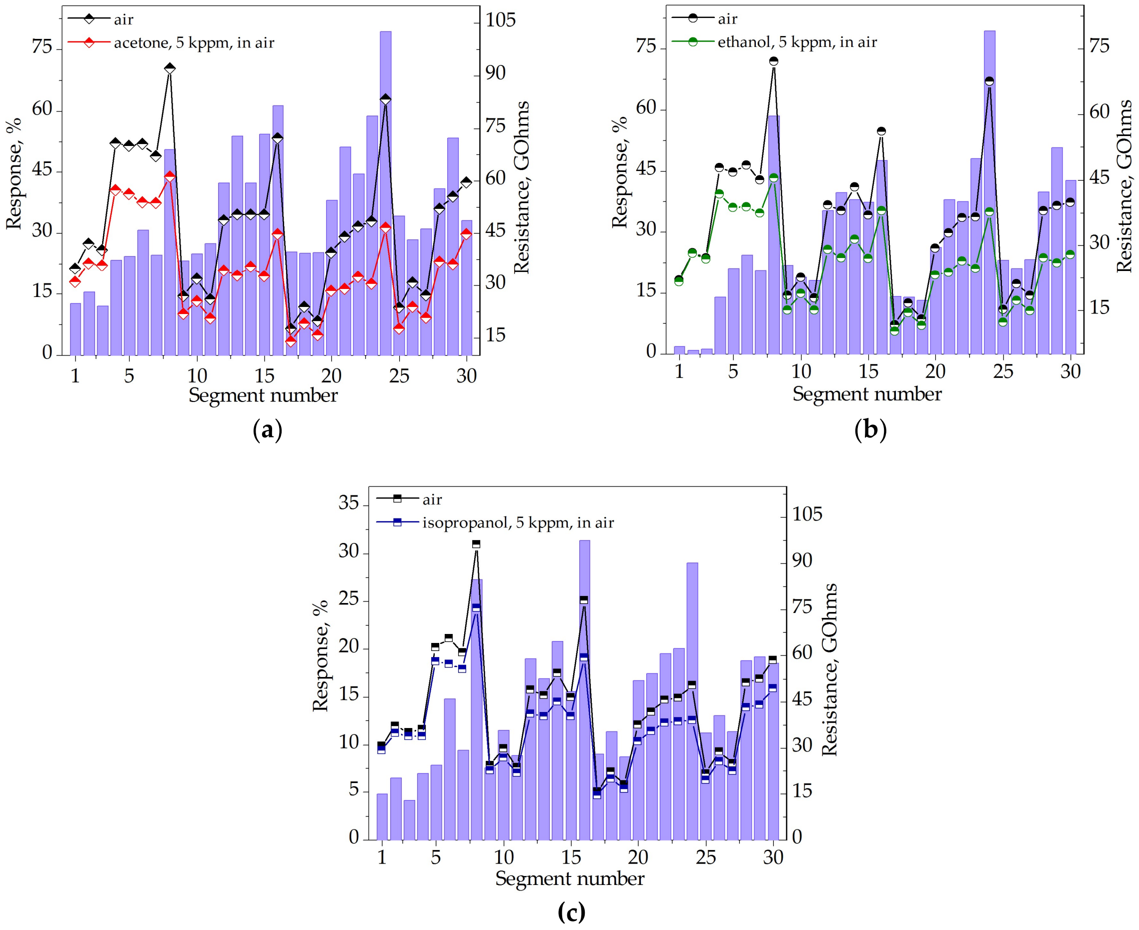

Figure C2. The gas response of the multisensor chip based on titanate whiskers towards organic vapors, ~ 5 kppm concentration, in the mixture with lab air at room temperature distributed over the chip array and corresponding conductance values at chip exposure to air and the vapors, acetone (**a**), ethanol (**b**), isopropanol (**c**) in the mixture with air.

Supplemental References

S1. Wen, P.; Itoh, H.; Tang, W.; Feng, Q. Transformation of layered titanate nanosheets into nanostructured porous titanium dioxide in polycation solution. *Microporous Mesoporous Mater.* **2008**, *116*, 147–156.

S2. Li, Q.; Kako, T.; Ye, J. Facile ion-exchanged synthesis of Sn^{2+} incorporated potassium titanate nanoribbons and their visible-light-responded photocatalytic activity. *Int. J. Hydrog. Energy* **2011,** 36, 4716–4723.

S3. Liu, Y.; Qi, T.; Zhang, Y. Synthesis of hexatitanate and titanium dioxide fibers by ion-exchange approach. *Mater. Res. Bull.* **2007**, *42*, 40–45.

S4. Jede, R.; Peters, H.; Dünnebier, G.; Ganschow, O.; Kaiser, U.; Seifert, K. Quantitative depth profile and bulk analysis with high dynamic range by electron gas sputtered neutral mass spectrometry. *J. Vacuum Sci. Technol. A* **1988**, *6*, 2271–2279.

S5. Goschnick, J.; Natzeck, C.; Sommer, M. Discrete shape analysis of the energy distribution to discriminate non-atomic signal contributions in depth profiling with SNMS. *Surf. Interface Anal.* **1999**, *28*, 56–60.

S6. Goschnick, J.; Sommer, M. Rapid soil analyses of overburden material from historic mines with SNMS, *Fresenius J Anal. Chem.* **1998**, *361*, 704–707.

S7. Goschnick, J.; Maeder, U.; Sommer, M. The depth distribution of organic additives in mortar measured with plasma-based secondary neutral mass spectrometry. *Fresenius J Anal. Chem.* **1998**, *361*, 707–709.

© 2017 by the authors. Submitted for possible open access publication under the terms and conditions of the Creative Commons Attribution (CC BY) license (http://creativecommons.org/licenses/by/4.0/).
